# Update on Targeted Therapy in Medullary Thyroid Cancer

**DOI:** 10.3389/fendo.2021.708949

**Published:** 2021-08-19

**Authors:** Christian Okafor, Julie Hogan, Margarita Raygada, Barbara J. Thomas, Srivandana Akshintala, John W. Glod, Jaydira Del Rivero

**Affiliations:** ^1^Pediatric Oncology Branch, Center for Cancer Research, National Cancer Institute, National Institutes of Health, Bethesda, MD, United States; ^2^Developmental Therapeutics Branch, Center for Cancer Research, National Cancer Institute, National Institutes of Health, Bethesda, MD, United States

**Keywords:** medullary thyroid cancer (MTC), MEN2, calcitonin, tyrosine kinase inhibitor, clinical trials

## Abstract

Medullary thyroid carcinoma (MTC) is a rare neuroendocrine tumor that accounts for 2-4% of all thyroid cancers. All inherited MTC and approximately 50% of sporadic cases are driven by mutations in the *RE*arranged during *T*ransfection (*RET)* proto-oncogene. The recent expansion of the armamentarium of RET-targeting tyrosine kinase inhibitors (TKIs) has provided effective options for systemic therapy for patients with metastatic and progressive disease. However, patients that develop resistant disease as well as those with other molecular drivers such as RAS have limited options. An improved understanding of mechanisms of resistance to TKIs as well as identification of novel therapeutic targets is needed to improve outcomes for patients with MTC.

## Background

Medullary thyroid carcinoma (MTC) is a rare neuroendocrine tumor that arises from the calcitonin-secreting parafollicular cells (C-cells). It has an estimated annual incidence ranging from 0.14 to 0.21/100,000 population and accounts for 2-4% of all cases of thyroid cancer (1–3). All reported cases of hereditary MTC as well as 40-50% of sporadic cases are attributed to activating mutations in the *RE*arranged during *T*ransfection (*RET)* proto-oncogene (3). The *RET* gene encodes a transmembrane receptor tyrosine kinase that regulates a wide variety of cell processes such as survival, proliferation, motility, and apoptosis. Activating mutations in RAS underly approximately 40% of non-*RET* mutated sporadic MTC with most of the remaining cases having no identified oncogenic driver (4, 5). While RET has proved to be an effective therapeutic target, resistant disease can develop in the face of RET-targeting tyrosine kinase inhibitors (TKIs) and options for systemic therapy of non-RET driven MTC are limited. We will review the development of RET-targeting TKIs and ongoing efforts to understand and circumvent mechanisms of resistance. We will also discuss new targets for the systemic therapy of MTC including novel molecular targets, immunotherapy, and the ongoing challenges of developing an effective method of targeting RAS-driven tumors.

## Initiation of Systemic Therapy

The optimal timing for initiation of TKI therapy is unclear. Currently, systemic therapy should be reserved for patients with clear radiographic progression of disease or those with symptomatic disease. There is no evidence that TKI therapy is curative and there have been no completed phase III studies comparing approved agents. It has been recommended that the choice of TKI usage be patient-centered with initiation of therapy depending on patient medical history, concomitant medications, comorbidities and tumor characteristics (6). While RET-targeting TKIs have provided clinical benefit to patients with MTC, the development of resistant disease can occur and the toxicity of both cabozantinib and vandetanib limit their use in patients with small volume, asymptomatic or indolent disease. ATA guidelines recommend considering initiation of TKI therapy for patients with radiographic evidence of disease progression or symptomatic disease (7). The National Comprehensive Cancer Network (NCCN) guidelines (V 3.2020) recommend disease monitoring for patients that are asymptomatic with treatment with TKIs considered for disease that is unresectable or progressing by RECIST criteria (8).

## Exploiting RET as a Therapeutic Target

### RET Signaling in MTC

**Figure 1:**
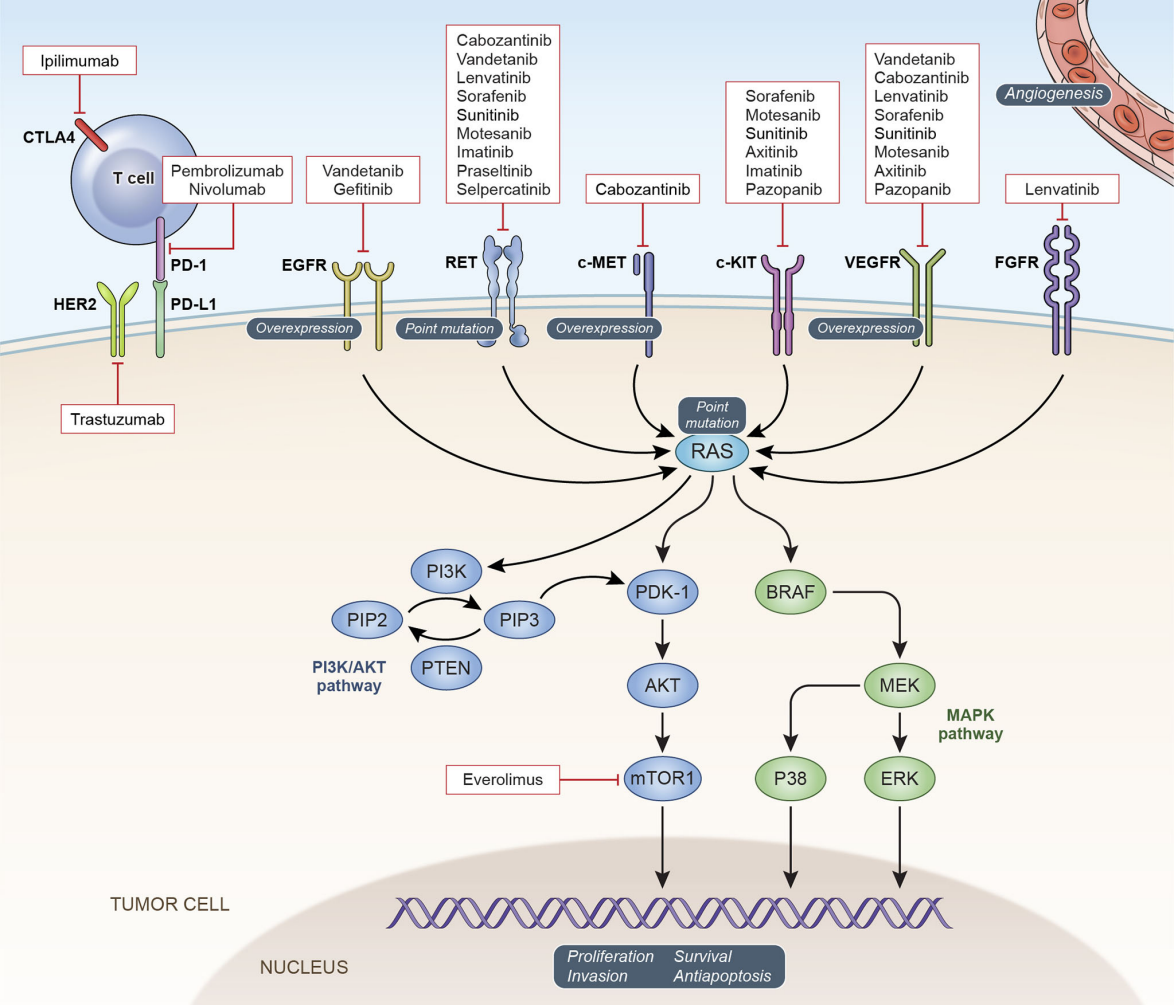
Oncogenic Signaling Pathways in MTC: Potential and confirmed therapeutic targets in MTC are depicted with currently available targeted agents.

The *RET* gene encodes a transmembrane receptor tyrosine kinase important in the regulation of cell growth, proliferation, migration, and survival (9). Physiological signaling through the RET receptor occurs upon binding of one of a family of glial cell-line derived neurotrophic factor (GDNF) ligands to a glycosylphosphatidylinositol-anchored coreceptor of the RET/GDNF family receptors alpha family (GFRα). This interaction leads to the formation of a dimeric GDNF-GFRα-RET complex and activation of the intracellular tyrosine kinase domains of both RET components of the dimer (10). (**Figure 1**) Physiological signaling through RET occurs following the phosphorylation of intracellular tyrosine residues on the RET protein that mediate the activation of multiple intracellular signaling pathways including PI3K/AKT, MAPK, JNK and others (10, 11). Oncogenic activating mutations of *RET* have been identified at several sites throughout the protein. Mutations in the cytosine-rich extracellular domain occur at codon C634 of exon 11 as well as 609, 611, 618, or 620 of exon 10. Mutation of these cysteine residues leads to formation of intermolecular disulfide bonds which result in covalent RET dimerization and kinase activation (12, 13). Germline mutations at these sites lead to MEN2A. The RET p.M918T mutation associated with MEN2B is in the intracellular tyrosine kinase domain 2, this amino acid change increases ATP binding affinity and leads to constitutive kinase activity in the absence of RET dimerization (12).

### Multi-Target Tyrosine Kinase Inhibitors

Disruption of RET signaling was first tested as a strategy for the treatment of patients with MTC using the multi-target kinase inhibitor, vandetanib (14). A 20% partial response rate was seen in patients treated on this phase II study. Vandetanib inhibits the activity of RET as well as other receptor tyrosine kinases including VEGFR-2, VEGFR-3, and EGFR (15) and the inhibition of each of these tyrosine kinases may play a role in the impact of vandetanib on MTC tumor growth. The drug was approved by the Food and Drug Administration (FDA) in April 2011 for the treatment of patients with symptomatic or progressive MTC with unresectable locally advanced or metastatic disease based on the results of a phase III trial which demonstrated a statistically significant improvement in 6-month progression-free survival (PFS) of 83% for patients treated with vandetanib compared to 63% for placebo (16). Importantly, the approval did not require the presence of a *RET* activating mutation. In 298 patients with sporadic MTC enrolled in this study a *RET* mutation was documented in 155 patients with no *RET* mutation in 8 patients and *RET* mutation status unknown in 135 patients. Cabozantinib, another multi-target TKI approved by the FDA for the treatment of patients with progressive or symptomatic MTC demonstrated an improved median PFS of 11.2 months compared to 4 months in a placebo group (17). A long-term follow-up analysis of this study after a minimum of 42 months did not show a statistically significant increase in overall survival with cabozantinib compared to placebo, although a non-significant 5.5-month increase was reported (18). A follow-up analysis showed an overall survival of 44.3 months in patients with tumors with RET M918T mutations treated with cabozantinib compared to 18.9 months for those treated with placebo. The difference in OS between cabozantinib and placebo treated patients who did not have M918T mutated tumors was 20.2 and 21.5 months (18). Because the cabozantinib study enrolled only patients with radiographic evidence of progressive disease compared to the initial phase 3 study evaluating vandetanib, which did not required evidence of disease progression for enrollment, it is difficult to compare the efficacy of the two agents. In addition to these studies in adult patients both vandetanib and cabozantinib were also effective for the treatment of pediatric patients with MTC (19–21). Despite these successes neither vandetanib nor cabozantinib leads to complete responses in patients with MTC and the development of resistant disease is a significant problem. As a consequence of their broad kinase inhibitor activity both of these agents have a number of toxicities. Toxicities seen with vandetanib included diarrhea, rash, nausea, hypertension, and headache (16) and patients who received cabozantinib, which inhibits hepatocyte growth factor receptor (MET) and VEGFR2 in addition to RET often experienced diarrhea, palmar-plantar erythrodysesthesia, decreased weight and appetite, nausea, and fatigue (17). The modest activity as well as the significant side-effects of multi-target TKIs can limit their utility in the treatment of patients with MTC.

Other multi-target TKIs have been investigated for the treatment of patients with MTC (**Table 1**). Sunitinib had an objective response rate of 38.5% with median PFS of 16.5 and OS of 29.4 months in a group of 26 patients (30). In a study of lenvatinib in 59 patients with MTC an objective response rate of 36% was reported (31). The trial included patients with both RET-driven and non-RET-driven disease and *RET* mutation status was not associated with response or PFS. Responses and clinical benefit have also been reported in patients with MTC with other multi-target TKIs including sorafenib (22, 32), and Anlotinib (23). Taken together, responses in patients with both RET and RAS-driven disease to TKIs with a range of targets suggests that the targeting of multiple signaling pathways could provide therapeutic opportunities for patients with advanced MTC. Additional TKIs including the multi-target agent regorafenib as well as next-generation RET-targeting TKIs are currently being evaluated (**Table 2**).

**Table 1:** Summary of results of completed tyrosine kinase inhibitor trials.

Tyrosine Kinase Inhibitors	Target(s)	Mutations	ORR	mPFS (months)	mOS (months)	Clinical Trial ID
**Vandetanib (** **22** **)**	VEGFR2-3, EGFR, RET	RET+RAS+UK	45%	30.5	NR	NCT00410761
**Cabozantinib (** **23** **)**	VEGFR2, KIT, FLT-3, RET, MET	RET+RAS+UK	28%	11.2	26.6	NCT00704730
		M918T Negative	20%	20.2	5.7	
		M918T	34%	13.9	44.3	
**Selpercatinib (** **24** **)**	RET, VEGFR2	RET/Previous TKI	69%	NR (1-year PFS 82%)	NR	NCT03157128
		RET/TKI Naïve	73%	NR (1-year PFS 92%)	NR	
**Sorafenib (** **25** **)**	BRAF, KIT, FLT-3, VEGFR2, PDGFR	ND	25%	NR	NR	NCT02114658
**Lenvatinib (** **26** **)**	VEGFR1-3, FGFR1-4, PDGFRα, KIT, RET	RET+RAS	36%	9	16.6	NCT00784303
**Anlotinib (** **27** **)**	VEGFR1-3, FGFR1-4, KIT FGFR	ND	48.4%	22.4	50.4	NCT02586350
**Pralsetinib (** **28** **)**	RET, VEGFR2	RET/Previous TKI	60%	NR	NR	NCT03037385
		RET/TKI Naïve	71%	NR	NR	
**Sunitinib (** **29** **)**	PDGFR, KIT VEGFR1-3, FLT-3, RET	ND	38.5%	16.5	29.4	NCT00510640

Completed trials with corresponding references are included. Mutation status is indicated as RET altered (RET), RAS altered (RAS), unknown (UK), and not determine (ND). Objective response rate (ORR), Progression free survival (PFS), and overall survival (OS) are indicated. NR indicates that median PFS or OS have not been reached.

**Table 2:** Ongoing early phase trials for patients with MTC.

Drug	Molecular Target	Phase	Eligibility	Clinical Trial ID
**Regorafenib**	BRAF, VAGFR1-3 PDGFRα/β, RET, KIT, FGFR1-2	2	MTC	NCT02657551
**TPX-0046**	RET	1/2	RET altered tumors	NCT04161391
**TAS0953/HM06**	RET	1/2	RET altered tumors	NCT04683250
**GFRα4 CAR T Cells**	GFRα4	1	MTC	NCT04877613

Currently open early phase studies including the multi-target TKI regorafenib as well as next generation RET-targeting TKIs and the first CAR T-cell targeting MTC.

### RET-Specific Tyrosine Kinase Inhibitors

The recently approved TKIs, selpercatinib and pralsetinib have more specific RET-targeting activity which leads to an improved side effect profile. In a phase 1-2 study of selpercatinib in patients with progressive *RET*-mutant MTC a 69% response rate was seen in patients who had previously received vandetanib or cabozantinib with a 73% response rate in TKI-naïve patients (33). The drug was well tolerated with grade 3 adverse events including hypertension (21%) and diarrhea (6%). Results of a study of pralsetinib in patients with MTC were recently reported with a response rate of 71% in TKI-naïve patients and 60% in patients who had previously received vandetanib or cabozantinib and pralsetinib was also FDA-approved for the treatment of patients with *RET* altered thyroid cancer (34). Selpercatinib was also effective and well-tolerated in five pediatric patients with cancers with activating mutations in *RET* including two patients with MTC (35). Selpercatinib and pralsetinib have several potential advantages compared to the multi-targeted TKIs including an improved side effect profile as well as activity against RET V804L and V804M mutants. However, resistance has also emerged to these newer TKIs. Solvent front mutations at RET glycine 810 as well as mutations at RET Y806 within the hinge region of the kinase cause resistance to both selpercatinib and pralsetinib (24, 36). Amplification of the *MET* gene has also been identified as a mechanism of resistance to selpercatinib in *RET* fusion non-small cell lung cancer (28). A combination of selpercatinib and the MET inhibitor crizotinib was able to overcome resistance due to *MET* amplification (28).

## Targets Downstream of RET

The success of RET inhibitors in the treatment of MTC suggests that downstream signaling pathways activated by RET may also be effective therapeutic targets. While the mechanisms that induce MTC oncogenesis through RET activation have not been completely defined, there is data supporting the involvement of a number of cellular signaling pathways in this process (**Figure 1**). The phosphatidylinositol 3-kinase (PI3K)/AKT/mammalian target of rapamycin (mTOR) pathway is activated in preclinical MTC models as well as cases of MTC (37, 38). This data as well as the efficacy of everolimus in other neuroendocrine tumors such as pancreatic NET led to its evaluation in patients with MTC. Responses have been reported in patients with MTC in several trials of the mTOR inhibitor everolimus (29, 39, 40). While dramatic response rates were not observed, this work provides additional evidence that the PI3K/AKT/mTOR pathway is important in MTC and may provide additional therapeutic targets for a subgroup of patients. Combined inhibition of both the RAS/MEK/ERK and PI3K/AKT/mTOR pathways has demonstrated activity in preclinical studies of MTC (25, 26). Unfortunately, combined inhibition of both pathways has thus far been limited by toxicity in early clinical trials (27, 41).

## RAS-Driven MTC

### RAS Mutant MTC

Approximately 70% of cases of MTC without an identifiable *RET* mutation have been attributed to *RAS* gene mutations (42–45). *RAS* genes code for a family of cellular signaling GTPases that regulate a wide range of downstream pathways including MEK/ERK and PI3K/AKT and play a role in numerous functions including cell growth and proliferation, apoptosis, and differentiation (45–47). Approximately 20% of all cancers have activating mutations in *RAS* (48). Activating mutations in *HRAS* are the most commonly seen in MTC with a lesser percentage of *KRAS* and rare *NRAS* mutations (44, 49, 50). *RAS* and *RET* mutations are typically mutually exclusive in MTC except for a small number of anecdotal reports with mutations in both genes (45). As with other RAS-driven cancers, the mutations seen in MTC occur primarily in exons 2 and 3 (45, 49). The most common activating mutations in *RAS* occur at G12, G13, and Q61 (51).

### Strategies for Targeting RAS

Targeting RAS and its associated signaling pathways has proven to be challenging. RAS has a lack of sites amenable to small molecule inhibitor binding and is difficult to target pharmacologically (52). RAS pathway inhibitors have been investigated for the treatment of MTC. In a phase I trial of the multi-kinase inhibitor sorafenib in combination with the farnesyltransferase inhibitor tipifarnib 5/13 patients with MTC showed a response, however the mutation status of the patients for *RET* and *RAS* was not reported (53). Post-hoc analysis was performed on data from a phase III trial of cabozantinib for MTC. Patients with cancers with mutations in *RAS* (*HRAS*, *KRAS*, and *NRAS*) had an overall response rate of 31% compared to a response rate of 32% in patients with *RET*-mutation positive disease with no survival benefit observed in patients with tumors lacking both *RET* and *RAS* mutations (54). Unlike KRAS and NRAS, HRAS is prenylated only by farnesyltransferase (55). The farnesyltransferase inhibitor tipifarnib is being tested in patients with HRAS driven cancers and patients with HRAS mutated MTC are eligible (NCT02383927).

Several novel strategies are currently being explored for the treatment of patients with *RAS* mutant cancers. A number of small molecule inhibitors of KRAS-G12C have recently been developed and are being clinically evaluated (56–58). Sotorasib and adagrasib are in phase II trials. Sotorasib was investigated in patients with cancers with KRAS-G12C mutation and had a disease response in approximately 30% of patients with non-small cell lung cancer (59). Other targeted inhibitors of specific mutated activated RAS isoforms are in clinical development (58). Activating mutations of *RAS* have also been targeted using immunological approaches. Infusion of expanded autologous KRAS-G12D-specific CD-8+ tumor infiltrating lymphocytes led to a response in a patient with *RAS* mutant colorectal carcinoma (60). Additional work has identified T-cell receptors that target activating mutations in KRAS (61–63). Based on this and other work early-stage trials are now underway using KRAS G12V and G12D-specific TCR cell therapy approaches (NCT04146298; NCT03190941; NCT03745326). While these approaches hold considerable promise, they are limited by specificity for a specific RAS isoform and mutation as well as the HLA restriction of TCR-based treatments.

## Immunotherapy and Other Targeted Therapies

### Peptide Receptor Radionuclide Therapy

Somatostatin receptor is present on a majority of cases of MTC (64) and can be detected with somatostatin receptor-based PET modalities (65). While no randomized controlled trials of Peptide Receptor Radionuclide Therapy (PRRT) in MTC have been done to date the use of radiolabeled somatostatin analogs for the treatment of MTC has been reported in a number of smaller studies. Patients treated with 90Y-DOTATOC had a response rate of 29% (9 of 31 patients) in a phase II study (66). In a single-institution retrospective analysis of patients with MTC treated with ^177^Lu-DOTATATE, a 62% response rate (27 of 43 patients) based on RECIST 1.1 criteria was reported (67). Recent reviews have summarized the published experience of PRRT in the treatment of patients with medullary thyroid cancer. Overall cumulative objective response rates to PRRT in patients with MTC of 5.1 and 10.6% were reported (68, 69). While these studies do show promise, the lack of controlled studies of PRRT in MTC as well as the potential for significant side-effects including hematologic and nephrotoxicity point to the need for randomized controlled trials in this area.

### Other Immunotherapeutic Approaches

Immunotherapy approaches are being tested for the treatment of patients with MTC including specific antigen-targeting approaches as well as strategies that leverage the anti-tumor immune response. Tumor vaccines incorporating the MTC secretory product carcinoembryonic antigen (CEA) have been studied in early phase clinical trials. An early study using vaccines generated with autologous mature dendritic cells loaded with calcitonin and CEA were studied in a small study of 7 patients. A decrease in calcitonin and CEA in 3/7 patients with one patient showing complete regression in metastatic nodules was reported in this trial (70). The CEA vaccine, GI-6207, was investigated in a phase 1 trial that enrolled 25 patients with metastatic CEA-expressing carcinomas and included one patient with MTC (71). The single patient with MTC on this study did not have a radiographic response, however, a marked inflammatory reaction at sites of metastatic disease was observed as well as significant T cell responses. Immune checkpoint inhibitors are also being explored in the treatment of MTC. A patient with recurrent MTC who was treated with a yeast-CEA cancer vaccine followed by anti-PD-L1 inhibitor, avelumab, was reported to have a 40% decrease in his calcitonin levels with radiographic stable disease. The tumor was found to express PD-L1 which may explain the calcitonin response to immunotherapy (72). Adoptive T-cell therapy has been successful in the treatment of patients with acute lymphoblastic leukemia and the strategy is being used to target other cancer types. Bhoj and colleagues reported preclinical data demonstrating that chimeric antigen receptor T-cells targeting GDNF family receptor alpha 4 could eliminate MTC in a murine xenograft model (73). An anti-GDNFα4 CAR T-Cell approach will be tested in a planned phase I trial (NCT04877613) (**Table 2**). An improved understanding of the immune microenvironment of MTC as well as identification of novel target antigens is needed to develop new effective immunotherapies for patients with MTC.

## Conclusion

The development of RET-targeting TKIs has made a substantial impact on the treatment of patients with MTC. Despite this advance, important challenges remain. Patients with sporadic MTC driven by *RAS* mutations as well as patients with disease resistant to the available RET-targeting TKIs have limited therapeutic options. Important questions remain in optimizing the use of TKIs in the treatment of patients with MTC. As use of the RET-specific TKIs increases it will be important to understand the patterns of resistance to these drugs and next-generation RET-targeting tyrosine kinase inhibitors that circumvent new resistance mutations will be needed. Improved understanding of downstream pathways involved in resistance, such as MET signaling, may inform the design of combination trials. Other novel strategies such as intermittent or alternating dosing of RET-inhibitors could also be considered. Another major challenge is the optimal timing for the initiation of TKI therapy. Large, randomized trials would be required to determine if initiation of a RET inhibitor prior to radiographic disease progression would provide any clinical benefit. RET-agnostic immunotherapy and antigen-targeting approaches also hold promise for expanding treatment options for MTC. While responses to PRRT have been reported, well-controlled prospective trials are still needed to define its role in the treatment of patients with MTC. T-cell based therapies have been highly effective in hematopoietic malignancies and the initial steps in their evaluation and development for the treatment of MTC are ongoing. These therapeutic strategies targeting specific antigens distinct from RET, if effective, would be important advances. Studies of these agents may also improve our understanding of the immunology of MTC as well as stimulating the search for additional targetable antigens in MTC. Metastatic MTC is currently a treatable but incurable cancer. However, rapid advances in multiple therapeutic strategies holds promise for an eventual cure for this disease.

## Author Contributions

CO prepared the initial draft of the manuscript and revised the manuscript. JG and JR critically reviewed and revised the manuscript. JH, MR, BT, and SA reviewed and revised the manuscript and provided input. All authors contributed to the article and approved the submitted version.

## Funding

This work was supported by the NCI My Pediatric and Adult Rare Tumor (MyPART) Network. The MyPART Network is a group of physicians, researchers, patients, their families, and advocates dedicated to finding treatments and improving the lives of people with rare solid tumors.

## Conflict of Interest

The authors declare that the research was conducted in the absence of any commercial or financial relationships that could be construed as a potential conflict of interest.

The reviewer MAZ declared a shared affiliation, though no other collaboration, with the authors to the handling Editor.

## Publisher’s Note

All claims expressed in this article are solely those of the authors and do not necessarily represent those of their affiliated organizations, or those of the publisher, the editors and the reviewers. Any product that may be evaluated in this article, or claim that may be made by its manufacturer, is not guaranteed or endorsed by the publisher.
